# OP82 The Opportunity Cost Of Funding High-Cost Medicines: Two Case Studies In Latin America

**DOI:** 10.1017/S0266462325101360

**Published:** 2025-12-29

**Authors:** Catalina Gutiérrez, Santiago Palacio, Ursula Giedion, Pamela Gongora, Lucia Bettati, Natalia Jorgensen, Marcella Distrutti, Dan Ollendorf

## Abstract

**Introduction:**

Like many Latin American countries, Colombia and the Dominican Republic grapple with the financial burden of covering high-cost medicines (HCMs). While some of these drugs are highly effective, others have reduced clinical effectiveness. Each of these drugs carries an opportunity cost in terms of the health gains lost by allocating these resources elsewhere. We quantified the health opportunity cost of funding HCMs in Colombia and the Dominican Republic.

**Methods:**

An identification strategy for HCMs was employed in both countries, based on their significant budgetary impact or high cost per case. This resulted in a shortlist of 10 molecules for Colombia and another 10 for the Dominican Republic. The health opportunity costs were estimated using the standard methodology of net health benefit, with the country-specific cost-effectiveness threshold employed as a proxy for average health production per dollar invested in the health systems of the two cases under study.

**Results:**

The selected HCMs included drugs for cancer, rare diseases, diabetes, and multiple sclerosis. The incremental health gains provided by these drugs were less than two life years in perfect health. In Colombia, funding the analyzed HCMs instead of the best available therapeutic alternatives implied an additional cost of USD642 million for the treatment of 22,155 covered patients. If these resources were allocated to expand services available within the system, the net gain would be 122,507 quality-adjusted life years (QALYs). In the Dominican Republic, the additional cost was estimated to be USD154 million (14,577 patients), with an estimated opportunity cost of 35,221 QALYs.

**Conclusions:**

The significant health opportunity costs for two countries with gaps in essential health services highlight the need for policies prioritizing efficient spending. Redirecting resources from the analyzed HCMs to cost-effective essential services could enhance health gains, addressing coverage gaps and advancing progress toward universal health coverage.

